# Gödelian embodied self-referential genomic intelligence: lessons for AI and AGI from the genomic blockchain

**DOI:** 10.3389/frobt.2025.1624695

**Published:** 2025-09-15

**Authors:** Sheri Markose

**Affiliations:** Department of Economics, University of Essex, Colchester, United Kingdom

**Keywords:** self-reference, Gödel sentence, blockchain, control or misalignment problem, genomic intelligence

## Abstract

The security of code-based digital records is a major concern of the 21st century. AI and artificial general intelligence (AGI) can be hacked to pieces by digital adversaries, and some AI objectives can lead to existential threats. The former arises from sitting duck problems that all software systems are vulnerable to, and the latter include control and misalignment problems. Blockchain technology, circa 2009, can address these problems: hashing algorithms rely on a consensus mechanism in manmade software systems to keep early blocks of software immutable and tamper-proof from digital malware, while new blocks can be added only if consistently aligned with original blocks. There is evidence that the ancient precedent of the genomic blockchain, underpinning the unbroken chain of life, uses a self-referential rather than a consensus-based hashing algorithm. Knowledge of self-codes permits biotic elements to achieve a hack-free agenda by self-reporting that they have been “negated,” or hacked, exactly implementing the Gödel sentence from foundational mathematics of Gödel, Turing, and Post (G–T–P). This results in an arms race in open-ended novelty to secure the primacy of original self-codes. Selfhood and autonomy are staples of neuroscience on complex self–other social cognition and increasingly of autonomous AGI agents capable of end-to-end programmed self-assembly. My perspective is that self-referential G–T–P information processing, first found in the adaptive immune system of jawed fish 500 mya and more recently in mirror neuron systems of humans, has enabled code-based self-organized intelligent systems like life to survive over 3.7 billion years. Some lessons for AGI can be gleaned from this discussion.

## 1 Introduction

Narrow artificial intelligence (AI) aimed at achieving specific tasks has had phenomenal success with large language models (LLMs), deep learning, and artificial neural network techniques based on multi-formatted data, including natural language, images, and numerical data. AI can surpass human competencies in tasks like pattern recognition, playing board games, and outputting text-based expert information in multiple domains, especially with LLMs. Some people are of the view that, as GPT-4 is capable of solving “novel and difficult tasks that span mathematics, coding, vision, medicine, law, psychology and more, without needing any special prompting,” (Bubeck et al., 2023), it already meets the hallmarks of artificial general intelligence (AGI). Jones and Bergen (2025) make similar claims for GPT-4.5, which aces the Turing test with a “win rate” of 73% of convincing human judges that the AI is human, while humans struggle to do so themselves. However, there has been pushback on AI acing Turing tests as being insufficient, or even a case of misdirected evidence of intelligence. Mitchell (2024) claims Turing tests suffer from moving goal posts due to “our shifting conceptions of intelligence.” The capacity of machines to hold fluent conversations in natural language that Turing proposed in 1950 is no longer considered to be evidence of general intelligence. Whether feats of GPT-4 LLMs qualify to be on par with human cognition, which marks an apogee for general intelligence, is part of ongoing debates (see Goertzel, 2014; Zimmermann, 2024).

Many have characterized human-level intelligence as having broad-ranging, adaptive powers that can respond to changing external environments by selecting goals and the means to achieve them by including novel solutions. For instance, having given a long list of characteristics of human-level intelligence, which includes self–other awareness and self-control, Goertzel (2014) requires AGI to have “general scope and is good at generalization across various goals and contexts.” With regard to novel solutions, to date, the open-ended adaptive capacity of humans produces what Dawkins (1989) calls extended phenotypes or artifacts outside of ourselves, rather than following a trans-human agenda with genomic enhancements.

It has become commonplace to state that intelligence is what mediates the goals–means nexus and is characteristic of goal-directed agents (Russell and Norvig, 2003). The pushback on GPT LLMs on having a Q&A format, in which the AI does not learn anything, due to insufficient experientially driven data from the environment actively elicited by the agent, has been raised by Silver and Sutton (2024). Their vision of the next stage of AI agents is of those that are autonomous in their selection of goals and means, capable of self-learning from a continuous stream of experientially driven feedback governed by reward maximization. However, we have here the infamous proclamation of Captain Ahab in Moby Dick, “All my means are sane, my motive and my object are mad.” This calls into question what “sane” goals are, if the only hallmark of rationality qua intelligence, in extant decision sciences (see Markose, 2024; Silver et al., 2021), is the reward maximization calculus of efficiency in the service of an objective.

In recent discussions, the AI control problem or the misalignment problem (Bostrom, 2014; Russell, 2019; Ngo et al., 2023; Hinton, 2023) has been recognized when AI systems are autonomous and evolve malign behaviors that may not align with human values and can evade human control. The Ngo et al. (2023) description of AI agents that use deception and power-seeking strategies to pursue misaligned goals underscores this as a perennial problem of political economy that is not unique to AI. At least since the Hobbesian thesis on the struggle for power and resources, it has been recognized that there is an existential threat to life and society when an agent with unbridled adaptive intelligence is free to set its own goals and encounters other similarly intelligent agents with their goals. The problem of adversarial and conflicting goals is writ large. The extant computational environment is swarming with sniffers, snipers, deep fakes, and computer viruses. In all cases, though these bots are installed by humans, they can operate with various degrees of autonomy to deceive, defraud, bring down software systems, and, in the case of killer bots and drones, physically decapitate humans and destroy their digital and material possessions. Generative adversarial networks (GANs), for instance, can program bots to resist detection by making deep fakes of themselves.

In this note, I aim to throw new light on three aspects of the control or misalignment problem of AGI. For this, I will draw on advances in gene, neuro, and computer sciences, especially cryptography, on how to protect purposeful software systems that can lose autonomy when malware agents can hack and hijack host codes to do their bidding. What is interesting is that autonomy and selfhood, often considered to be vestiges of liberal democracy, are part of the unique information processing of a code-based system of life that has maintained the unbroken chain of life while permitting evolutionary change.

The first step is to refer to the above discussions, the provenance of general intelligence as a means of maintaining homeostasis of life (Friston, 2010; Friston, 2013). In other words, the fundamental alignment of general intelligence is in the service of life itself and not any narrow objective. However, I will replace the Friston et al. Free Energy principle for self-organization of life’s homeostasis in terms of minimizing the degrading forces of entropy and disorder with a code-based explanation for general intelligence. I will elaborate on how the digital socio-economic world driven by AI has parallels with what I call genomic intelligence (Markose, 2024), which accords with the Walker and Davis (2013) epigram on the “algorithmic take-over” of biology with digitization of inheritable information encoded in a near-universal alphabet (A, T, C, G/U) in the genome.

Second, I will introduce the reader to the adversarial digital game, coextensive with life itself as the fundamental source of misalignment, that was brought to my attention by the game theorist Ken Binmore (see Markose, 2021c). Binmore (1987) raised the specter of Gödel’s Liar, qua digital adversary, who will negate what can be predicted. Binmore uses Gödel’s Liar to highlight the flaw of extant Game Theory: by confining the best response to a given action set, Game Theory not only guarantees that determinism will be punished by the Liar but also precludes novelty and surprises in the Nash equilibrium of a game. Markose (2017) produces a Nash equilibrium of a game with Gödel’s Liar, which, from logical necessity, produces novel syntactic objects outside listable sets. To date, complexity, evolvability, novelty production, and “thinking outside the box” in biology and humans have, for the most part, relied on models of randomness or on statistical white noise error terms (Markose, 2024; Markose, 2021b). This is despite the long-standing type IV dynamics in the Wolfram–Chomsky schema, based on foundational mathematics of Gödel, Turing, and Post (G–T–P) aka recursion function theory (RFT), that only code-based computational systems that can embrace self-referential recursive structures of the Gödel incompleteness theorems (GITs) can produce novelty (see Prokopenko et al., 2019; 2025). Given that for some 90 years there has been little evidence that GITs and the capstone construction in the form of the Gödel sentence has relevance to any real world phenomena, in Section 2, I will unpack some of the recent evidence of how such self-referential intelligence was acquired for complexification over the course of evolution of multicellular eukaryote life (Markose, 2022).

Third, a major development of the 21st-century digital age, which has a bearing on the misalignment problem, is the astounding invention of the blockchain distributed ledger technology (BCDL). This was first presented in the anarchic agenda of Bitcoin by pseudonymous Nakamoto (2008) to resist centralized state control of monetary systems. BCDL permits decentralized software-based record keeping of actions of multiple agents, in which the fidelity of extant digital records is maintained by a hashing solution to a cryptographic puzzle. This also makes it difficult for malign activity regarding new software additions by a subset of agents. Abramov et al. (2021), Markose (2021a), and Markose (2022) have been the first to point out that the genome is a blockchain. However, while Abramov et al. (2021) utilize the consensus mechanism well known in manmade blockchain (Hussein et al., 2023). Markose (2022) indicates that the genomic blockchain relies on a self-referential hashing solution using the Gödel sentence, which permits biotic elements to self-report that they are under attack. The immutability of protein coding blocks of life for 3.7 billion years, associated with Crick’s notion of a “frozen accident” while novelty is added, in the 21st century, can be identified as part of a unique self-referential BCDL embodied in the organism that secures alignment with the homeostasis of life. In any case, there is a growing understanding that unless software systems are embedded in a BCDL, they will be doomed to failure by optimization of narrow objectives, as in the Bostrom (2014) paperclip apocalypse, or hacked to pieces due to the sitting duck problem (see Nabben, 2021; Heaven, 2019).

## 2 Staples of G–T–P/RFT, genomic intelligence, and homeostasis of life

Until recently, there has been little evidence of how the staples of RFT and Gödel (1931) relate to genomic systems, let alone to BCDLs. This section will unpack the breakthroughs on the evidence that RFT staples are ubiquitous in the self-referential genomic intelligence of eukaryotes.

### 2.1 Unique digital identifiers and hashes in biology

First, which is also a major ingredient of BCDLs for malware detection, is the feature of unique digital identifiers pioneered by Gödel (1931), called Gödel numbers (g.n.) or indexes, whereby a finite string of letters maps to a unique integer. The hash compresses variable-length strings to a fixed length, and any change in input strings will alter the hash. There is now extensive evidence of bio-peptide and other unique identifiers, including “zip codes” for cellular signal processing, as discovered in the Nobel prize-winning work of Blobel (1999). It appears that all signaling in bio-ICT relies on peptide identifiers from transcription factors in gene expression to neuron-neuron links. As in the design of BCDLs that all nodes of the distributed system have the same information, more than 30 trillion cells in a human have the same DNA, with some exceptions of mosaicism. There is evidence (see Brickner et al., 2012) that subnetworks of gene regulatory networks have characteristic identifiable binding motifs in transcription factors and their binding sites for associated gene expression for temporal and specialized cell development in tissues. We will denote by *g, g ∈ G,* the DNA instructions that lead to gene expression of specific somatic and phenotype developments of the organism, where *G* is the set of expressed genes.

### 2.2 Self-reference and diagonal self-assembly machines in biology

In RFT, using epithets from Hofstader (1999), we have self-reference (Self-Ref) or diagonal operators typically stated as a program, *g*, that builds a machine that runs *g* and halts (denoted as *ɸ*_*g*_ *(g)* ↓). Gershenfeld (2012) and Gershenfeld et al. (2017) give the remarkable insight that what 21st-century digital fabrication aims to do, which is described as end-to-end code-based 3-D self-assembly of digitized materials, is something biology solved 3.7 billion years ago. The self-assembly programs of biology are associated with the ribosome and other transcriptase machinery that implement gene expression for the morphological, somatic identity, and regulatory control of the organism.

**FIGURE 1:**
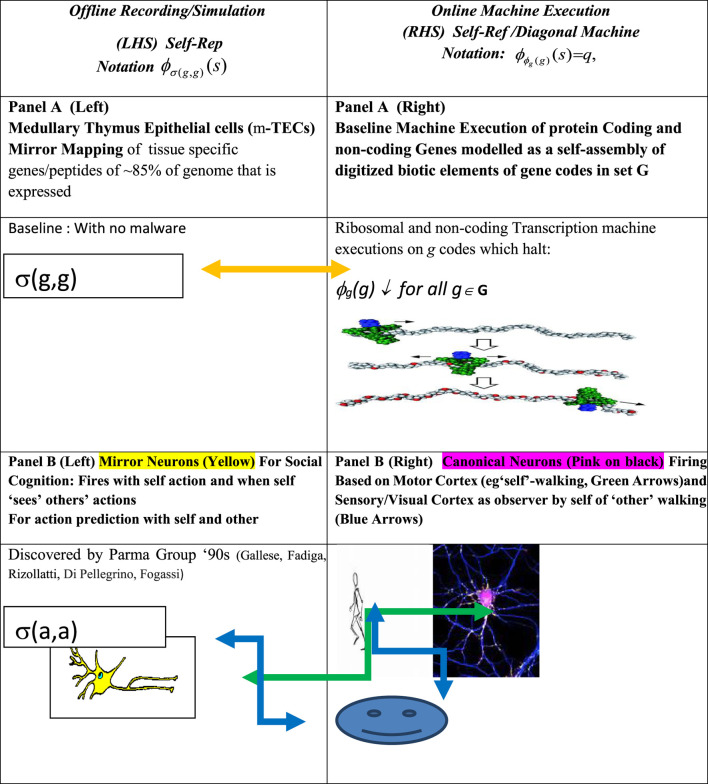
Gödel meta-representation (Rogers, 1967) and mirror systems in immuno-cognitive systems. Note: *Offline* mirror systems in the medulla thymus (Panel A, Left) and *Offline* cognitive mirror neuron system (Panel B, Left) and a respective bijective map of *Online* gene transcription (Panel A, Right) and *Online* action execution in the motor–sensory cortex (Panel B, Right).

The breakthrough on the significance of this staple of self-referential/diagonal operator in RFT found in textbooks like Rogers (1967) and Cutland (1980) for biological self-assembly is given in Panel A of Figure 1. Following the set theoretic proof of GITs in Post (1944), Cutland (1980), and Smullyan (1961), *g ∈ G,* that determine selfhood of the organism can be considered the theorems for the organism and G–T–P information processing, and alignment to the homeostasis of life is stringently governed by the principle of consistency.

### 2.3 Offline self-representation (Self-Rep) or mirror mappings of online self-assembly machine executions

A major breakthrough here is the evidence Markose (2021a), Markose (2022) found for Self-Rep mirror structures of the adaptive immune system (AIS), approximately 500 mya post jawed fish, which is not present in prokaryotes. The major histocompatibility complex (MHC1) receptors of the thymus are found to record 85% of expressed genes relating to the 3D self-assembly of the morphology and somatic identity of the organism. This is shown, respectively, in the left (offline recording) and right (online self-assembly Self-Ref machine execution) sides of Panel A of Figure 1. For good reason, these self-repped gene codes in the thymus have been called the *Thymic Self*, Sánchez-Ramón and Faure (2020) or “the science of self” Greenen (2021). As is well known, the Self-Rep in AIS thymic receptors is primarily to identify the hostile other, viz., negation function operators of non-self-antigens, as will be discussed below. Indeed, Miller et al. (2019) wax lyrical: “As self-referential cognition is demonstrated by all living organisms, life can be equated with the sustenance of cellular homeostasis in the continuous defense of ‘self’.” This is remarkable in that Miller et. al. give centrality to self-referential information processing in genomic systems specifically to detect and mitigate adversarial changes to self-codes but make no reference to the RFT staple of the Gödel representation theorem from Rogers (1967), which is exactly depicted in Figure 1. As a result, Miller et al. (2019) is a compendium of analogies and possible inaccuracies but lacks RFT or a code-based explanation of how self-referential structures implement the defense of self-codes.

An even larger literature in neuroscience on mirror mappings has followed important discoveries of the Parma Group (Fadiga et al., 1995; Gallese et al., 1996; Rizzolatti et al., 1996; Gallese and Sinigaglia, 2011; Gallese, 2009) of a mirror neuron system (MNS) in the primate brain where embodied self-action codes from the sensory-motor cortex are mapped *offline* and reused to make action inference in conspecifics and help facilitate complex self–other interactions (see also Markose et al. (2025)). However, despite the central role assigned to self-reference for the sentient self in advanced organisms (Gardenfors 2003; Northoff et al., 2006; Newen, 2018; Miller et al., 2019, etc.), only Tsuda (2014), Markose (2017), Markose (2021b), and Markose (2022) have noted how the evolutionary development of Self-Rep offline mirror structures is necessary for biotic elements to make statements about themselves. Tsuda (2014) makes an explicit observation that unless the two-step mirror Self-Rep recursive structures are in place, the mapping between the online machine execution codes and the offline recording of the same shown in Figure 1 using the σ (x,x) 2 – place, the Gödel substitution function, it is unlikely that statements about self can be made, let alone about the other.

### 2.4 How can changes to genomic self-codes be detected, specifically those brought about by a digital adversary?

Here, the breakthrough in gene science, which debunks the idea that the primary source of evolutionary changes arises from random transcription/replication errors, follows the epochal discovery by Nobel Laureate Barbara McClintock (1984) of transposable elements (TEs) of viral origin. TEs that conduct cut-paste (transposons) and copy-paste (retrotransposons) give a code-based explanation for genomic changes. TEs, which account for some 45% of the genome, have been found to engineer genomic evolvability, brain plasticity, and novel phenotypes primarily in eukaryotes (Fedoroff, 2012). This underscores the truism that only software can change software and also sheds light on the double-edged sword of viral software. It can benignly copy and paste as in replication, which entails a simple sliver of code, as shown in one of the earliest accounts of code biology by Kauffman (2015). However, malign viral hacking, done externally by bio-malware and or internally by TEs to gene expression itself, forms the Achilles heel of genomic digital systems.

Hence, here we have the model for the self-referential detection of Gödel’s Liar. This entails the adaptive immune system (AIS) (Flajnik and Kasahara, 2009) in the T-cell receptors that “simulate” the application of negation software functions, *f* ^*¬*^, qua virus (hacker) on self-repped gene codes. The breakthrough here is to see that an RFT generalization of Gödel (1931) using Roger’s Fixed Point Theorem (Rogers,1967) is needed for the counterparts in the periphery of the self-repped gene codes in the T-cell receptors to self-report when software changes to self-codes are brought about by novel non-self-antigens in real time. The latter are an uncountable infinity.

The AIS implements “out of the box” astronomic anticipative search for novel non-self-antigens necessary for novel antibody production and cognition in humans, manifesting unbounded proteanism for novel extended phenotypes (Dawkins, 1989) in the form of artifacts outside of ourselves. This facility, first found in the AIS, relies on the recombination activator genes (RAG 1 and 2) and also in the human brain for neural receptor diversity (Muotri et al., 2009; Kaesar and Chun, 2020; Peña de Ortiz and Arshavsk, 2001), which runs into orders of magnitude of 10^20^–10^30^ (Kapitonov and Jurka, 2005) that exceed the pre-scripted germline of the genome size many times over. Likewise, detection of negation of what is predicted in the human mirror neuron system found in neuroscience experiments by Scott Kelso and co-authors (Tognoli et al., 2007) gives evidence for perception of deceit and complex counterfactuals in the Theory of Mind in social cognition.

The Rogers (1967) fixed point indexes of the Second Recursion Theorem for yet-to-happen *f* ^*¬*^ attacks by the non-self-antigens are generated in the AIS in a most ingenious fashion: a large number of codes/indexes purported to be of different *f* ^*¬*^ on each self-repped *g* are generated in the T-cell receptors. This is the most spectacular case of predictive coding. Suppose that the g.n for the tuple { *f* ^*¬*^, *g* } specifying that *f* ^*¬*^ has attacked *g*, is denoted by *g* ^*¬*^. When the attack by *f* ^*¬*^ takes place in real time in the periphery involving *g*, the experientially driven peripheral MHC1 receptor mediated by interferon gamma must record this. If this “syncs” with the one that was speculatively generated in the thymic MHC1 receptors, two parts of the fixed point come together to construct a genomic Gödel sentence, which will now have a fixed-point index of σ (*g* ^*¬*^ *, g* ^*¬*^ *).* At this point, *g* self-reports that it is under attack.

The index σ (*g* ^*¬*^ *, g* ^*¬*^ *)* of the Gödel sentence effectively signals the hash for an untenable state of 0 = 1 produced by the fixed point of a *f* ^*¬*^ negation function of self-codes (see Kauffman, 2023). Such syntactic objects, σ (*g* ^*¬*^ *, g* ^*¬*^ *)* at the point at which it is recursively generated, are undecidable in that they lie outside of listable sets arising from the mapped self-repped expressed gene codes that are the theorems for the organism and the list of indexes for known non-theorems. Such indexes σ (*g* ^*¬*^ *, g* ^*¬*^ *)* of Gödel sentences have recently been identified by Markose (2017), Markose (2021a), and Markose (2022) as a precursor for endogenous novelty production in genomic systems. Indeed, it is a testable hypothesis that it is the inability of the peripheral MHC1 receptor to update the index to σ (*g* ^*¬*^ *, g* ^*¬*^ *)* when the *f* ^*¬+*^attacks *g*, typically due to faulty interferon gamma mediation, that causes AIS to fail to generate novel antibodies (Markose, 2021a). In RFT, the productive set of Post (1944) provides the unique recursive construction of the blockchain of fixed point indexes σ (*g* ^*¬*^ *, g* ^*¬*^ *)* for the novel non-self-antigens and the novel antibodies thereof. This takes on the structure of an arms race, which is somatic and *extraneous* to the germline; hence, this exercise is geared to conserve the genome rather than to improve it.

Schmidhuber (2006) and more recent articles (Zhang et al., 2025) have depicted Gödel machines and Darwin Gödel machines, respectively, to show how self-referential mappings can lead to self-improving machines that can rewrite their own codes. It is important to note here that the precise implementation of the structures of Gödel incompleteness as found in the adaptive immune system, which involves the detection of novel negation functions of adversarial agents and their fixed-point indexes as in the Gödel sentence, the novel antibody production that follows does not lead to self-improvement in the germline. Instead, the self-referential recursive structures are geared toward conserving self-codes against adversaries, and the arms race in novelty is to improve defenses and maintain autonomy of the organism against prolific digital adversaries.

It is conjectured that an identical RFT machinery is involved in the self–other nexus in both the AIS and MNS. What evidence is there for this? In a knockout of interferon gamma in the Jonathan Kipnis Group experiment on rats (Filiano et al., 2016), it was found that the rats lost immune capabilities as well as their social cognition of recognizing another rat. Kipnis et al. give an Evo-Devo explanation that evolution has taught rats to socially isolate when their immune system is compromised. My code-based explanation (see Markose, 2021a) is that the same self-referential recursive structures are in place *both* for the AIS as well as in the brain MNS for self–other cognition and hence when the interferon gamma mediator, especially in the *peripheral* MHC1 receptor is knocked out, the circuitry for the fixed point generation needed for predictive coding for non-self–other misfires and self becomes blind to the other. It is conjectured that this is how the rats in the Filiano et al. (2016) experiment lost their immune capabilities *and* their capacity for social cognition of another rat.

## 3 Concluding remarks

In conclusion, genomic intelligence in vertebrates that has reached its pinnacle in humans is highly empathic as the conspecific/other is the projection of self; greatly Machiavellian having co-evolved from adversarial viral agents; geared toward unbounded proteanism from the get-go starting with transposon-based diversity creation by recombination activation genes (RAG) in the immune system and brain; and stringently self-regulated by a self-referential block chain distributed ledger (BCDL) driven by the principle of autonomy of the life of the organism and an agenda to be hack free.

It is a matter of incredulity that some 90 years have passed since Gödel (1931), for evidence to be found that the RFT staples of Self-Ref and Self-Rep and the Gödel sentence are ubiquitous in biology and genomic intelligence. Several factors can be adduced for the lack of precise computational modeling of self-reference in the context of general intelligence. Even those who espouse that code-based operations are relevant in cognition, such as in the Computational Theory of Mind (see Rescorla, 2020), never mention Self-Ref, self-assembly machines, Self-Rep mirror systems, or computational fixed-point indexes and, of course, the role of the Gödel sentence. There is a strong anti-machine view that claims that biology is a non-digital “natural” process that is creative in some vitalistic way. This view overlooks the fact that in nature, only biology, with the encoded basis of the genome, and the extended phenotypes of humans who have built computers, manifest software-based digital systems.

Two canards are associated with the Gödel incompleteness theorems (GITs) that seem to propagate anti-machine vitalistic beliefs about life and intelligence. These have posed a stumbling block to the necessary breakthroughs on code-based explanations for genomic information processing. The canards are that the GIT proves that human cognition is not computational and self-reference leads to paradox (see Battaglia et al., 2025). In the Gödelian setting, as unlike the Cretan Liar paradox *This is False*, Gödel’s painstaking two-step process of Self-Ref and Self-Rep, found in Rogers (1967) generalizations thereof, on how statements about self and other appear to be made in the immune-cognitive systems (see Figure 1; Markose, 2021b), there are no paradoxes. Furthermore, influential commentators like Roger Penrose have used the GIT to conclude that human cognition can outstrip what Turing machines can do. As Rescorla (2020) says, “It may turn out that certain human mental capacities outstrip Turing-computability, but Gödel’s incompleteness theorems provide no reason to anticipate that outcome.” The work of LaForte et al. (1998), *On Why Gödel’s Theorem Cannot Refute Computationalism,* and others has provided push back on such flawed anti-machine views on biology and human cognition.

Section 2 gives an account of how G–T–P based immune-cognitive systems may be conducting self-referential information processing. As first noted by Tsuda (2014), it is unlikely that any statements regarding self or the other can be made by humans without the two-step Self-Ref and Self-Rep recursive information processing structures having evolved. Furthermore, the recursive generation of an index of the Gödel sentence should demystify what it is, a hash representing “0 = 1,” viz., non-theoremhood or misalignment, and how it signifies a novel object as a constructive “witness” for proof of incompleteness. In view of this, the following statement is misconstrued: “The paradox of a brain trying to study itself presents a conundrum, raising questions about self-reference, consciousness, psychiatric disorders, and the boundaries of scientific inquiry” (Battaglia et al., 2025). Likewise, in the absence of the precise recursive function structures of Self-Ref and Self-Rep necessary to identify software changes to self-codes, the important discovery by the Parma Group of the mirror neuron system in the brain has been stymied by hype and inaccuracies.

In the influential non-code-based Free Energy principle explanation for general intelligence involved in the homeostasis of life (Friston, 2013), Friston does not fall into the trap of the mainstream optimization framework, which effectively constrains search to under the lamp post and cannot produce novelty. Schwartenbeck et. al (2013) state that search for novel solutions and “explorative behavior is not just in accordance with the principle of free energy minimization but is, in fact, mandated by it.” However, from the vantage of the discussion here, it seems that there has been insufficient consideration by Friston of the regulatory framework of maintaining homeostasis of life’s vital signs within feasible physical/analog states, viz., minimizing “surprisals,” when this is under the aegis of smart algorithmic controls. The latter must contend with software-related data security breaches from bio-malware or adversarial digital agents.

What has been overlooked is that a large part of homeostasis in formalistic code-based self-assembly systems of life involves the complexification of phenotype with dynamic adversarial digital game structures that must embrace an arms race in novelty and surprises in order to avoid threats to autonomy from adversarial agents that can hack gene codes. This is a problem that genomic intelligence appears to have solved. AI, in contrast, has ignored this self-referential design for data integrity for autonomous existence that can vitiate what is called the “sitting duck” problem (Heaven, 2019). Furthermore, extant decision sciences are devoid of any epistemic structures for novelty production and complexification (Markose, 2024). As noted, there is a considerable difference between the Gödel self-reference models for novelty production by Markose (2021c), Markose (2022) and those of Schmidhuber (2006) and Zhang et al. (2025). I underscore the formal system premise of consistency and theoremhood for providing the stringent selection mechanism for what novelty is permitted in genomic systems and do not use the language of optimal self-improvement or reward frameworks as do these and other authors.

In closing, it is my view that the biological immuno-cognitive model of the self-referential genomic BCDL with Gödel sentence hashes has far-reaching implications for understanding the full gamut of self–other pathologies, gene regulatory networks that must deal with malign transposable element activity, and more robust design solutions for sustainable AGI.

## Data Availability

The original contributions presented in the study are included in the article/supplementary material; further inquiries can be directed to the corresponding author.
